# Unveiling the hidden struggle of healthcare students as second victims through a systematic review

**DOI:** 10.1186/s12909-024-05336-y

**Published:** 2024-04-08

**Authors:** José Joaquín Mira, Valerie Matarredona, Susanna Tella, Paulo Sousa, Vanessa Ribeiro Neves, Reinhard Strametz, Adriana López-Pineda

**Affiliations:** 1 Atenea Research. FISABIO, Alicante, Spain; 2 Universidad Miguel Hernández, Elche, Spain; 3 Faculty of Health and Social Care, LAB University of Applied Sciences, Lappeenranta, Finland; 4 NOVA National School of Public Health, Public Health Research Centre, Comprehensive Health Research Center, CHRC, NOVA University Lisbon, Lisbon, Portugal; 5 Escola Paulista de Enfermagem, Universidade Federal de São Paulo, São Paulo, Brasil; 6 Wiesbaden Institute for Healthcare Economics and Patient Safety (WiHelP), RheinMain UAS, Wiesbaden, Germany

**Keywords:** Adverse events, Patient safety, Resilience, Second victims, Students

## Abstract

**Background:**

When healthcare students witness, engage in, or are involved in an adverse event, it often leads to a second victim experience, impacting their mental well-being and influencing their future professional practice. This study aimed to describe the efforts, methods, and outcomes of interventions to help students in healthcare disciplines cope with the emotional experience of being involved in or witnessing a mistake causing harm to a patient during their clerkships or training.

**Methods:**

This systematic review followed the PRISMA guidelines and includes the synthesis of eighteen studies, published in diverse languages from 2011 to 2023, identified from the databases MEDLINE, EMBASE, SCOPUS and APS PsycInfo. PICO method was used for constructing a research question and formulating eligibility criteria. The selection process was conducted through Rayyan. Titles and abstracts of were independently screened by two authors. The critical appraisal tools of the Joanna Briggs Institute was used to assess the risk of bias of the included studies.

**Results:**

A total of 1354 studies were retrieved, 18 met the eligibility criteria. Most studies were conducted in the USA. Various educational interventions along with learning how to prevent mistakes, and resilience training were described. In some cases, this experience contributed to the student personal growth. Psychological support in the aftermath of adverse events was scattered.

**Conclusion:**

Ensuring healthcare students’ resilience should be a fundamental part of their training. Interventions to train them to address the second victim phenomenon during their clerkships are scarce, scattered, and do not yield conclusive results on identifying what is most effective and what is not.

**Supplementary Information:**

The online version contains supplementary material available at 10.1186/s12909-024-05336-y.

## Introduction

Students in healthcare disciplines often witness or personally face stressful clinical events during their practical training [1, 2], such as unexpected patient deaths, discussions with patients' families or among healthcare team members, violence toward professionals, or inappropriate treatment toward themselves. When this occurs, the majority of students talk to other students about it (approximately 90%), and less frequently, they speak to healthcare team members or mentors (37%) [2]. This is because they usually believe they will not receive attention, will not be understood, or fear negative consequences in their evaluation [1, 2].

A particular case of a stressful clinical event is being involved in an adverse event (AE) or making an honest mistake [2] due to circumstances beyond the student's control. Approximately three-quarters of nursing or medical students witness some AE during their professional development (clerkships and training in healthcare centers) [2, 3] and studies show that 18%-30% of students report committing an error resulting in an AE [4, 5]. Some of them may even experience humiliation or verbal abuse for that error [6]. The vast majority (85%) of these occurrences lead to a second victim experience [7, 8]. Consistent with what we know about the second victim experience [9–11], it is common for students in these cases to experience hypervigilance, acute stress, and doubts about their own ability for this work [12, 13]. These emotional disturbances are usually more intense among females than males [14] and people with high values in the personality trait of neuroticism [15, 16].

They also observe the impact of clinical errors on other healthcare professionals, influencing their response [3]. All these situations affect their well-being and can shape their future professional practice style [17, 18]. For example, they may develop defensive practices more frequently [5, 17] or avoid informing patients in the future after an AE [4]. Educators should not overlook the emotional effects of AEs on students/trainees [19]. Indeed, patient safety topics, including the second victim, mental well-being, and resilience, are neglected in undergraduate medical and nursing curricula in Europe. Furthermore, over half (56%) according to the responses from the students they did not ‘speak up' during a critical situation when they felt they could or should have [20].

Recently, psychological interventions to promote resilience in students facing stressful situations have been reviewed [21]. These interventions are not widely implemented, and approximately only one-fourth of students report having sufficient resilience training during their educational period [2]. In the specific case of supporting students who experience the second victim phenomenon, we lack information about the approach, scope, and method of possible interventions. The objective of this systematic review was to describe the efforts, methods, and outcomes of interventions to help students in healthcare disciplines cope with the emotional experience (second victim) of being involved in or witnessing a mistake causing harm to a patient during their clerkships or training.

## Methods

The review was conducted following the Preferred Reporting Items for Systematic Reviews and Meta-Analyses (PRISMA) guidelines [22]. The study protocol was registered at PROSPERO (International prospective register of systematic reviews) [23] under the registration number CRD42023442014.

### Eligibility criteria

The research question and eligibility criteria were constructed using the PICO method as follows (see Supplemental material 1):

- Population: Students of healthcare disciplines
- Intervention: Any method or intervention addressing the second victim phenomenon
- Comparator: If applicable, any other method or intervention
- Outcomes: Any measure of impact

Eligible studies included those reporting any method or intervention to prevent and address the second victim experience among healthcare students involved in or witnessing a mistake causing adverse events during their clerkships or training. Additionally, studies reporting interventions addressing psychological stress or reinforcing competences to face highly stressful situations, enhancing resilience, or increasing understanding of honest errors in the clinical setting were also included. Regarding the study population, eligible studies included healthcare discipline students (e.g., medical, nursing, pharmacy students) enrolled in any year, level, or course, both in public and private schools or faculties worldwide. All quantitative studies (experimental, quasi-experimental, case–control, cohort, and cross-sectional studies) within the scope of educational activities, as well as all qualitative studies (e.g., focus groups, interviews) conducted to explore intervention outcomes, were included.

The exclusion criteria were interventions and data regarding residents or professionals as trainees, analysis aimed at preparing the curriculum content or evaluating academic performance (including regarding patient safety issues), and any type of review study, editorials, letters to the editor, comments, or other noncitable articles (such as editorials, book reviews, gr*ey* literature, opinion articles or abstracts). Conference abstracts were included if they contained substantial and original information not found elsewhere.

### Search

The search was conducted on August 5, 2023, in the following electronic databases: MEDLINE, EMBASE, SCOPUS and APS PsycInfo. The reference lists of relevant reviews and other selected articles were explored further to find any additional appropriate articles. Last, recommended websites (gray literature) found during the comprehensive reading of publications were included if they met the inclusion criteria.

Controlled vocabulary and free text were combined using Boolean operators and filters to develop the search strategy (Supplemental Material 1). The terminology used in this study was extracted from the literature while respecting the most common usage of the terms prior to initiation of this screening. No limitations were imposed regarding language or the publication date.

### Study selection

The selection process was conducted through Rayyan [24]. After removal of duplicates, two researchers (JM and VM) independently screened the titles and abstracts of all retrieved publications to determine eligibility. Discrepancies were resolved by an arbiter (AL), who made the final decision after debate to obtain consensus. Afterwards, screening of the full texts of the preselected articles was carried out in the same manner.

### Data extraction

After final inclusion, the following characteristics of each study were collected by two reviewers: publication details (first author, year of publication), country of the study location, aim/s, study design, setting, type of study participants, and sample size. Separately, the following information of the included studies was collected: the description of methods, support programs or study interventions to address the second victim phenomenon, the findings on their effectiveness (competences and attitudes changed) and participants’ views or experience, if applicable, and whether a ‘second victim’ term was used.

### Quality appraisal

We used the critical appraisal tools of the Joanna Briggs Institute [25] to assess the risk of bias of the included studies, according to the study design. Those studies that did not meet at least 60% of the criteria [26] were considered to have a high risk of bias. The critical appraisal was performed by two independent reviewers, and the overall result was expressed as a percentage of items answered with “yes”. Additionally, the number of citations of each article was collected as a quality measure [27].

### Data synthesis

A descriptive narrative synthesis of the studies (approaches and outcomes) was conducted comparing the type and content of the methods or interventions implemented. Before initiating our literature search, we drafted a thematic framework informed by our research objectives, anticipating potential themes. This framework guided our evidence synthesis, dynamically adapting as we analyzed the included studies. Our approach allowed systematic integration of findings into coherent themes, ensuring our narrative synthesis was both grounded in evidence and reflective of our initial thematic expectations, providing a nuanced understanding of the topic within the existing research context. All data collected from the data extraction were reported and summarized in tables. The main findings were categorized into broad themes: (1) Are students informed about the phenomenon of second victims or how to act in case of making a mistake or witnessing a mistake? (2) What do students learn about an honest mistake, intentional errors, and key elements of safety culture? (3) What kind of support do students value and receive to manage the second victim phenomenon? (4) Strategies for supporting students in coping with the second victim phenomenon after making or witnessing a mistake. We considered the effectiveness (measurement of the achieved change in knowledge, skills, or attitudes) and meaningfulness (individual experience, viewpoints, convictions, and understandings of the participants) of each intervention or support program.

## Results

**Fig. 1:**
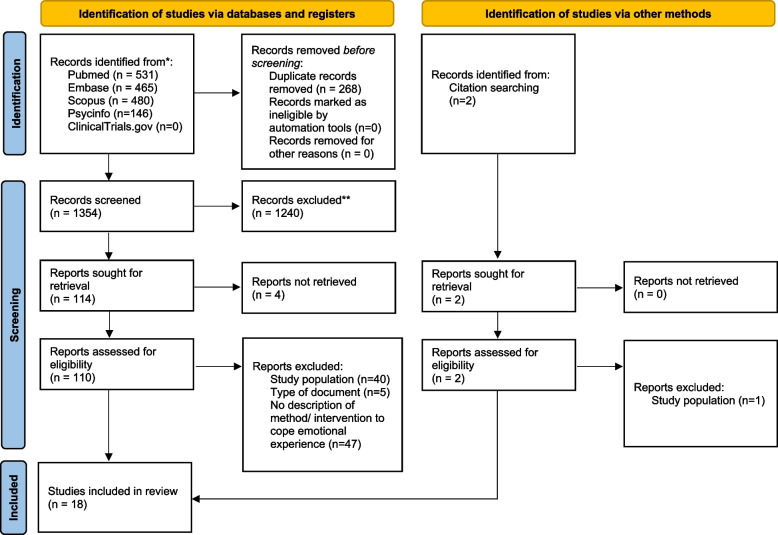
PRISMA 2020 flow diagram for new systematic reviews which included searches of databases, registers and other sources

A total of 1622 titles were identified after the initial search. After removing duplicates, 1354 studies were screened. After the title, abstract and full text review, we identified and extracted information from 18 studies. The selection process is shown in the PRISMA flow diagram (Fig. 1).

The articles included in this review are shown in Table 1 in alphabetical order of the first author, detailing the characteristics and overall result of the quality assessment (measured as the percentage of compliance with the JBI tool criteria) of each study. Most studies were conducted in the USA (*n* = 7) [19, 21, 28–32], followed by Korea (*n* = 2) [33, 34] and Australia (*n* = 2) [35, 36], and the rest were carried out in Denmark [37], China [38], Italy [39], the United Kingdom [40], Georgia [41], Brazil [42], and Canada [43] (*n* = 1 each). The included studies cover a publication period that ranges from 2011 to 2023, with four of them being published in 2020. All these investigations were conducted within the academic setting, with the exception of one study, which took place in the Western Sydney Local Health District. Regarding the study participants, eleven studies were exclusively focused on medical students, six specifically targeted nursing students, and one included both medical and nursing students. In terms of study design, quasi-experimental (*n* = 8), cross-sectional (*n* = 2) and qualitative designs (*n* = 6) were used, and two studies used a mixed-methods design.

**Table 1:** Characteristics and quality assessment results of the included studies (*n* = 18)

First author’ name, year of publication	Country of the study location	Aims	Study design	Setting	Type of study participants	Sample size	Overall result of the quality assessment	Citations^a^
Breslin A, 2019 [28]	USA	To develop, implement, and evaluate a shame resilience after medical error seminar	Quasi-experimental (pre–post-test survey)	Duke University School of Medicine	Fourth year medical students	179	55.5%	0
Davis M, 2022 [29]	USA	To offer a high-reliability organization (HRO)-related medication safety curriculum to nursing students and to evaluate their learning	Quasi-experimental (pre–post-test survey)	Community college	Senior associate degree nursing students	53	66.7%	5
Gillies RA, 2011 [41]	Georgia	1. To develop and evaluate a multi-faceted intervention for teaching medical students about medical errors and apologies 2. To know the opinion of the participants about the intervention	1. Quasi-experimental (pre–post-test survey) 2. Cross-sectional (survey study)	Medical College of Georgia	First-year medical students	384	55.5%	11
Hanson J, 2020[35]	Australia	To know the students' opinion about the quality, effectiveness and appropriateness of a communicative assertiveness activity	Qualitative study (survey with qualitative questions and individual semi-structured interview)	School of Nursing, Midwifery and Paramedicine, at a regional university in South East Queensland	First year undergraduate nursing students	535	80%	28
Huang H, 2020 [38]	China	To explore the degree of second victim syndrome in clinical practice and the rehabilitation process	Mixed-methods design (quantitative survey and a semi-structured interview)	Medical schools of China	Nursing students	147 (survey study) 6 (interview)	80%	10
Kim CW, 2017 [33]	South Korea	To assess the effectiveness of an education program for medical error disclosure	Quasi-experimental (pre–post-test survey)	Seoul National University College of Medicine (SNUCM), Seoul National University Hospital (SNUH), Chung-Ang University Hospital (CUH)	Fourth-year medical students	79	66.7%	18
Krogh, TB. 2023 [37]	Denmark	To explore experiences, perceptions, and management of second victim phenomenon	Qualitative study (semi-structured focus group Interviews)	University of Copenhagen	Medical students and recent medical graduates (not starting the 1st year of training)	15	80%	0
Lane AS, 2021 [36]	Australia	To examine the experience of students about learning of open disclosure, using high fidelity mannequins and human actors, in the context of medication error’	Qualitative Study (focus groups)	Western Sydney Local Health District	Final-year medical students of a 4-year postgraduate medical programme	8	70%	3
Le H, 2022 [30]	USA	To design and implement an educational experience to cope with medical errors To know the opinion of participants about the importance of medical error, and the feeling of comfort when they discuss medical errors	Cross-sectional (survey study)		4th year medical students			0
Mohsin SU, 2019 [44]	USA	To develop an intervention for helping students to identify and report a medical error, and to describe the impact on clinical error reporting	Quasi-experimental (pre–post-test survey)	Wayne State University School of Medicine	Medical students	282	55.5%	15
Mousinho TAP, 2022 [42]	Brazil	To identify the support provided to nursing students after a patient safety incident	Qualitative study (interviews and survey)	Federal university in Rio Grande do Sul	Last year nursing students	23	70%	
Musunur S, 2020 [31]	USA	To develop an educational session to allow students to discuss the impact of medical error with local physician-educators in their community and prepare them before the first adverse event occurs To assess the impact of this curriculum	Quasi-experimental (pre–post-test survey)	Wayne State University School of Medicine (Detroit)	Second year medical students	300	66.7%	2
Noland CM, 2015 [32]	USA	To explore how students understand medical errors and how they communicate about those errors	Qualitative Study (Interview)	New England University	Nursing students	68	80%	12
Rinaldi C, 2022 [39]	Italy	1-To describe the prevalence of second victim 2-To explore the physical and psychological symptoms after a patient safety incident, the causes and the received support	Cross-sectional (survey study)	University of the Piemonte Orientale (Novara)	Nursing students (*n* = 128) and medical students (*n* = 174)	302	75%	2
Roh H, 2014 [34]	Republic of Korea	To examine the changes in the perceptions (concepts of patient safety), in the attitudes focusing on systems failure rather than blaming individuals for medical errors, and the individual and collective responsibility in medical students after a patient safety education	Quasi-experimental (pre–post-test survey)	Inje University College of Medicine	Third-year medical students	98	66.7%	24
Ryder HF, 2019 [19]	USA	To develop, implement, and evaluate the patient safety reporting curriculum (PSRC)	Quasi-experimental (pre–post-test survey)	The Geisel School of Medicine at Dartmouth	Medical Students	126	55.5%	4
Thomas I, 2015 [40]	UK	To develop and evaluate a simulated ward round experience with a focus on medical error and distraction	Mixed-method survey study	University of Aberdeen	Final-year medical students	28	70%	7
Zieber MP, 2015 [43]	Canada	To explore the experience of nursing students who had made at least one mistake in their clinical practice	Qualitative Study (semi-structured interview)	Nursing school of Canada	Third or fourth year nursing students	16	80%	9

^a^October 24, 2023

Supplementary Tables 1, 2 and 3 show the quality assessment of quasi-experimental, cross-sectional, and qualitative studies, respectively. Four of the included studies [19, 28, 41, 44] did not meet at least 60% of items and were considered to have a high risk of bias. The five studies of highest quality [32, 35, 37, 38, 43] met 80% of the items. The study of Le et al. (2022) [30] did not have enough information to assess the risk of bias, as it was a conference abstract. The study cited the most is the Hanson et al. study, conducted in 2020 [35].

Table 2 shows educational interventions, support strategies and any method reported in the scientific literature to help healthcare students cope with the emotional experience (second victim) of being involved in or witnessing a mistake during their clerkships or training. Due to the heterogeneity of retrieved studies regarding the type of design, the intervention type and outcome measures, a statistical analysis of the dataset was not possible. Thus, the evidence was summarized in broad themes.

**Table 2:** Methods, support programs and interventions to address the emotional experience (second victim) of being involved in or witnessing a mistake causing harm to a patient during their clerkships or training

First author, year	Methods to address the SV phenomenon	Outcome measure results (if applicable)	Use of second victim term
Breslin A, 2019 [28]	2.5-h seminars consisting of: 1. a large group session to introduce the psychology of shame and guilt responses to medical error 2. current residents shared personal narratives of shame experiences encountered during medical training 3. students met in small groups to discuss their reactions to the large-group content	Significant increasement (*p* < 0.001) in confidence in identifying shame, in ability to differentiate shame from guilt, to identify shame reactions, and reported increased willingness to ask for help from others	No
Davis M, 2020 [29]	A 3-h interactive lecture/discussion session which consists of 6 HRO modules adapted from the World Health Organization Patient Safety Curriculum. Each module opened with an anecdote (video or narrative format), which depicted an actual episode of patient care error relevant to that module's topic	After the educational intervention, participants improved pretest to posttest scores about their knowledge, application skills, and critical thinking by 74%. They evaluated the learning experience positively	Yes
Gillies RA, 2011 [41]	Multi-faceted apologies intervention using Miller’s clinical competence pyramid as a model (ie, learners move from knowledge to competence, performance, and action). These tasks included online reading and interactive apology tasks to small-group and standardized patient interactions	Perceived utility of the course and module: 66% considered useful or extremely useful Perceived utility of apology evaluations: 74% considered useful or extremely useful Perceived utility of standardized patient interview: 62% considered useful or extremely useful Increased confidence in providing effective apologies Increased their comfort in disclosing errors to a faculty member or patient Increased perceived importance of apology skills	No
Hanson J, 2020 [35]	Preparatory framework for ‘speaking up for safety’ To use the rehearsal and practice of a graded assertiveness technique using the P.A.C.E and C.U.S·S frameworks in a preparation for their practice course	Reaching assertiveness skills and establishing a preparatory framework for ‘speaking up for safety’ early in a nursing students tertiary education can have important psychosocial implications for their confidence, empowerment and success	No
Huang H, 2020 [38]	Discussing the patient safety incident with professionals or peers Support and understanding of patients	Not applicable	Yes
Kim CW, 2017 [33]	Education program that included practice of error disclosure using a standardized patient scenario, debriefing session with clinical vignettes describing medical error. feedback, and short didactic sessions	Participants’ abilities to disclose medical error: 65% of participants said that they had become more confident in coping with medical errors, and most participants (79.7%) were satisfied with the education program	No
Krogh T, 2023 [37]	Current coping strategies: Individual and social processing, either using more formal offers of support (voluntary or mandatory) or their informal network	Not applicable	Yes
Lane AS,2021 [36]	4-h education session (simulation activity) based on open disclosure after medication error	Not applicable	
Le H, 2022 [30]	Educational intervention including: 1. a brief lecture on medical errors, 2. participation in a standardized patient encounter in which the students were required to disclose a medical error to the spouse of a critically ill patient 3. group debriefing focused on the challenges of disclosing medical errors and the impact of error on professional identity	94% agreed that medical error is an important topic 92% felt more comfortable discussing medical errors	No
Mohsin SU, 2019 [44]	4-h workshop including: 1. overview of patient safety and medical errors 2. episode of a TV show about a patient safety incident 3. overview on error analysis and tools 4. discussions about conditions contributing to errors, types of errors, error prevention, interventions/actions and strength of actions to prevent errors 5. discussions about the importance of reporting: students were required to submit a simulated error report about an error they personally observed	Increased clinical error reporting frequency	No
Mousinho Tavares AP, 2022 [42]	Support from classmates and professors of the practical courses provided support The students showed no knowledge of organizational support or protocols available to students who become second victims of patient safety incidents	Not applicable	Yes
Musunur S, 2020 [31]	An hour-long interactive session, delivered by local faculty, which aims to show likelihood of being involved in a medical error, the professional and personal impact of medical errors through small group discussion and storytelling, and enabling students to identify both informational resources and individual personnel available at the local level if they or their colleagues faced with a medical error	Increased awareness of available resources in coping with medical errors Increased self-reported confidence in detecting and coping with medical error Pre-existing attitudes and knowledge regarding medical error stayed consistent	Yes
Noland CM, 2015 [32]	-To report mistakes and tell someone -Formal training in-class modules showing how to report a mistake -Informal education by the sharing of stories while in their clinicals (e.g. advice from a nurse) -Training about how to behave (remain calm) and to talk about the error with the patient and the supervisor -Training in Situation–Background–Assessment–Recommendation (SBAR) communication, as “a strategy to optimally prepare student nurses to communicate effectively within the clinical setting”	Not applicable	No
Rinaldi C, 2022 [39]	To talk to someone about the patient safety incident mainly with their colleagues, friends, clinical tutors, nurses, their partners, patients or patient’s relatives Formal support sources: the University Counseling Service and the General Practitioner Clinic	Not applicable	Yes
Roh H, 2014 [34]	Three-day patient safety course based on the World Health Organization (WHO) patient-safety guide for medical schools and previous research on patient safety education. The training consisted of: 1.basic concepts of patient safety (interactive lecture with video clips) 2. Error causes and quality improvements 3. Self-regulation and clerkship ethics 4. Teamwork and error-reporting 5. Communication with patients and caregivers 6. Frequent issues Using these methods: Interactive lecture with demonstration or videos, discussion with experts, small group practice, role-playing, practice with standardized patient, and debriefing)	Understanding, attitudes, and sense of responsibility regarding patient safety improved after training	No
Ryder HF, 2019 [19]	Interactive patient safety reporting curriculum (PSRC) to provide students with direct experience identifying, analyzing, and reporting medical errors This consisted of writing a structured written report, analyzing a patient safety incident they experienced The report focused on severity of outcome, root cause(s) analysis, system-based prevention, and personal reflection. The report was bookended by 2 interactive, case-based sessions led by faculty with expertise in patient safety, quality improvement, and medical errors	After the PSRC, students self-reported improved attitudes toward medical error and increased comfort with analyzing and disclosing them. Baseline attitudes remained high and significantly increased relative to historical controls Students receiving the PSRC in the second half of their third year reported higher levels of skill acquisition than students receiving training in the first half of their third year	No
Thomas I, 2015 [40]	A 30–minute simulated ward round experience with a focus on medical error and distraction	Students though that this simulated experience help them to reflect on positive behavioral changes for safe future practice, built confidence and was deemed to be of high fidelity. All students felt that mandatory curricular integration was important	No
Zieber MP, 2015 [43]	Support from peers, clinical instructor, family members,	Not applicable	No

### Are students informed about the phenomenon of second victims or how to act in case of making a mistake or witnessing a mistake?

Some authors focus on the identification and reporting of errors, assuming that this process helps to cope with the emotional experience after the safety incident. Their studies [19, 33, 34, 41, 44] reported information on trainings given to medical or nursing students based on how to disclose errors, without addressing the second victim phenomenon specifically. In 2011, Gillies et al. reported that a medical error apology intervention increased confidence in providing effective apologies and their comfort in disclosing errors to a faculty member or patient [41]. It included online content with interactive tasks, small-group tasks and discussion, a standardized patient interview, and anonymous feedback by peers on written apologies. In 2015, Roh et al. showed that understanding, attitudes, and sense of responsibility regarding patient safety improved after a three-day patient safety training. This study involved medical students who were instructed on error causes, error reporting, communication with patients and caregivers and other concepts of patient safety. They used interactive lectures with demonstrations, small group practices, role playing, and debriefing [34]. In 2019, Ryder et al. reported that an interactive Patient Safety Reporting Curriculum (PSRC) seems to improve attitudes toward medical errors and increase comfort with disclosing them [19]. This curriculum was developed to be integrated into the third-year internal medicine clerkship during an 8-week clinical experience. It aimed to enable students to identify medical errors and report them using a format similar to official reports. Students were instructed in the method of classifying AEs developed by Robert Wachter and James Reason's Swiss cheese model [12, 45]. A 60-min session included demonstrating the system model of error through a focused case-based writing assignment and discussion. In 2019, Mohsin et al. showed that clinical error reporting increased after a 4-h workshop where in addition to other concepts, the importance of reporting errors was discussed [42]. Other authors [30, 33] focused on students' ability to report these AEs with curricula and syllabi employing methods such as the use of standardized patients, facilitated reflection, feedback, and short didactics for summarization. These studies also reported that this type of education program seems to enhance students’ current knowledge [36] and abilities to disclose medical errors [30, 33].

Only the educational intervention suggested by David et al. in 2020, based on the World Health Organization (WHO) Patient Safety Curriculum, addresses the consequences and effects of the second victim phenomenon [29]. A 3-h session that consisted of the presentation of an AE in the form of a video or narrative, a discussion of case studies in small groups, where students have the opportunity to share their personal experiences related to these situations, and a list of practical application measures such as conclusions, improved knowledge, application skills, and critical thinking of students.

### What do students learn about honest and intentional errors and key elements of safety culture?

Most training for both medical and nursing students focuses on how to identify the occurrence of a medical error since students, when asked about it, show little confidence in their ability to recognize such errors because they are little exposed to clinical procedures during their learning, which makes it difficult for them to differentiate errors from normal practice. In addition to teaching them how to identify them, interventions have also focused on how to prevent these AEs before they happen, as well as how to talk about them once they occur [29, 40, 41, 44]. None of the training mentioned in the studies included in this review incorporated education on honest or intentional errors. However, a patient safety curriculum for medical students designed by Roh et al. (2015) [34] and a medication safety science curriculum developed by Davis & Coviello (2020) [29] for nursing students were based on the WHO Patient Safety Curriculum [13], which includes key aspects such as patient safety awareness, effective communication, teamwork and collaboration, safety culture, and safe medication management.

### What kind of support do students value and receive to manage the second victim phenomenon?

Students stated that the greatest support comes from their peers, followed by their mentors and, finally, their families and friends [32, 37–39, 42, 43]. Most hospitals and some universities have support programs specifically tailored for such situations, offering psychological assistance [39]. However, as these are mostly voluntary aids, many students do not make use of them, and if they do, the support they receive is usually limited. Mousinho Tavares et al. (2022) found that the students did not know about the organizational support or protocols available to students who become second victims of patient safety incidents [42]. In 2020, in the USA, interactive sessions exploring the professional and personal effects of medical errors were designed to explain to medical students the support resources available to them [31].

### Strategies for supporting students after making or witnessing a mistake

In 2019, Breslin et al. developed a 2.5-h seminar on resilience for fourth-year medical students (in the USA) consisting of an initial group discussion about the psychology of shame and the guilt responses that arise from medical error [28]. During this first group discussion, students had the opportunity to share their experiences related to these concepts encountered during their medical training. Following this, students formed small groups led by previously trained teachers to enhance their confidence in discussing shame and to further explore the topics covered in the group seminar. This training improved confidence in recognizing shame, distinguishing it from guilt, identifying shame reactions, and being willing to seek help from others. In 2020, Musunur et al. showed that an hour-long interactive group session for medical students in the USA increased awareness of available resources in coping with medical errors and self-reported confidence in detecting and coping with medical errors [31]. A 2022 Italian cross-sectional study on healthcare students and medical residents as second victims found no data on structured programs included in medical residency programs/specialization schools to support residents after the occurrence of an adverse event. The study also found that it might be interesting to design interventions for posttraumatic stress disorder (PTSD) for this type of student, as the symptoms of second victims are similar to those of this disorder. Similarly, this study proposes a series of interventions that could be useful, such as psychological therapy, self-help programs, and even drug therapies, as they have been proven effective in treating PTSD [39].

## Discussion

Few training interventions exist to support healthcare students cope with emotional experiences of being involved in or witnessing a mistake causing harm to a patient during their clerkships. These interventions are scattered and not widely available. Additionally, there's uncertainty about their effectiveness.

In 2008, Martinez and Lo [3] highlighted that during students' studies, there are numerous missed opportunities to instruct them on how to respond to and learn from errors. This study seems to confirm this statement. Despite some positive published experiences, the provision of this type of training is limited. Deans, school directors, academic and clinical mentors, along with faculty members, have the opportunity to recognize the needs of their students, helping to prepare them for psychologically challenging situations. Such events occur frequently and are managed by professionals who rely on their own capacity for resilience. These sources of stress are not unknown to us, as they are a regular part of daily practice in healthcare settings. However, they do not always receive the necessary attention, and it is often assumed that they are addressed without difficulty [3].

Currently, we are aware that students also undergo the second victim experience [8, 37, 46], and it has been emphasized that this experience may impact their future professional careers and personal lives [39]. There is a wide diversity in training programs and local regulations regarding the activities that students in practice can undertake. Although there is a growing interest, the number of studies has increased since 2019, there are still many topics to address, and the extent of the experiences suggests that these are isolated initiatives without further development informed in other faculties or schools.

Over one-third of the studies have employed quasi-experimental designs with pre-post measures, although most studies have relied on qualitative methodologies to explore students' responses to specific issues [19, 28, 29, 31, 33, 34, 41, 44]. These investigations do allow us to assert that we understand the problem, have quantified it, and have ideas to address it, but we lack a consensus-based and tested framework to ensure the capacity to confront these situations in the students. Moreover, similar to what occurs in the study of training in resilience or to face the second victim phenomenon in the case of healthcare workers [2, 21, 28, 35, 47, 48], all of the studies have been focused on medical and nursing students. Other profiles (such as pharmacy or psychology students) have not been included until now.

The first study on the impact of unintended incidents on students in healthcare disciplines dates back to 2011. Patey et al. (in 2007) identified deficiencies in patient safety training among medical students and designed an additional training module alongside their educational program [6]. Other experiences have also focused on providing patient safety education [6, 29, 33, 35, 39].

The majority of studies included in this review focused on training students in providing information and apologies to patients who have experienced an AE (due to a clinical error). These studies have been conducted on every continent except Africa, and while they have different objectives, they share a similar focus: enhancing the skills to disclosure and altering defensive or concealment attitudes. Many students had difficulty speaking up about medical errors [49]. This fact poses a threat to patient safety. The early formative period is the optimal time to address this issue, provide skills, and overcome the traditional and natural barriers to discussing things that go wrong.

Students preparing for highly stressful situations in their future careers face a contrast between the interest in their readiness and the observed figures of clinical errors during practices. A 2010 study [37] in Denmark reported that practically all students (93% of 229) witnessed medical errors, with 62% contributing to them. In Belgium (thirteen years after), up to 85% of students witness mistakes [17], while US and Italy studies (2019–2022) showed lower figures. Among 282 American students, only 36% experienced AEs, and Italian nursing students reported up to 37% [4, 8, 10]. Students are witnessing 3.8 incidents every 10 days [48], although there are students who do not report witnessing any errors during their clinical placements, indicating difficulties with speaking up. Preparing students for emotional responses and reactions from their environment when an adverse event occurs seems necessary in light of these data.

Although the information is limited (a total of 125 students were involved), the data provided by Haglund et al. (in 2009) suggest that being involved in highly stressful situations contributes to reinforcing resilience and represents an opportunity for their personal growth [48]. Training to confront these stressful situations, including clinical errors, helps reduce reactive responses, although it does not guarantee maintaining the previous level of emotional well-being among students [21]. In this sense, the model proposed by Seys et al. [50], which defines 5 stages, with the first two focused on preventing second victim symptoms and ensuring self-care capacity (at the individual and team levels), could also be applied to the case of students and, by extension, to first-year residents to enhance their capacity to cope with an experience as a second victim.

AEs are often attributed to professional errors, perpetuating a blame culture in healthcare [51]. Students may adopt defensive attitudes, risking patient safety. Up to 47% [4] feel unprepared for assigned tasks, and 80% expect more support than received [39]. Emotional responses to EAs include fear, shame, anxiety, stress, loneliness, and moral distress [1, 5, 14, 17, 20, 21],. Students face loss of psychological well-being, self-confidence, skills, job satisfaction, and high hypervigilance [10, 13, 17]. While distress diminishes over time, mistakes' impact may persist, especially if harm occurs [5]. Near misses can positively contribute to education, raising awareness. of patient safety [52]. Simulating situations using virtual reality enhances coping abilities and indirectly improves patient safety [53].

In spite of these data, students are typically not informed about the phenomenon of second victims or how to respond in the event of making or witnessing a mistake, including during their period of training in faculties and schools [54]. They express a desire for support from their workplace and believe that preparation for these situations should commence during their university education [4]. Students attribute errors to individual causes rather than factors beyond their control (considering them as intentional rather than honest mistakes). There have been instances of successful experiences demonstrating how this information can be effectively communicated and students can be equipped to deal with these stressful situations. Notably, there are training programs aimed at enhancing disclosure skills among medical and nursing students [33, 36]. However, the dissemination of such educational packages in faculties and schools is currently limited. This study was unable to locate research where the concepts of honest or intentional errors were shared with students.

Support interventions for second victims should provide a distinct perspective on addressing safety issues, incorporating the principles of a just culture, and offering emotional support to healthcare professionals and teams, ultimately benefiting patients. These interventions have primarily been developed and implemented within hospital settings [55]. However, comprehensive studies are lacking, and experiences within schools and faculties, as well as extending support to students during their clinical placements, appear to be quite limited. Conversely, there exists a body of literature discussing the encounters of residents from various disciplines when they assume the role of second victims [38]. These experiences should be considered when designing support programs in schools and faculties. In fact, a recent study has described how students seem to cope with mistakes by separating the personal from the professional and seeking support from their social network [37]. Models such as SLIPPS (Shared Learning from Practice to Improve Patient Safety) is a tool for collecting learning events associated with patient safety from students or other implementers. This could prove beneficial in acquainting students with the concept of the second victim phenomenon. Interventions in progress to support residents when they become second victims from their early training years could be extended to faculties and schools to reduce the emotional impact of witnessing or being involved in a severe clinical error [56]. However, it is essential not to forget that healthcare professionals work in multidisciplinary teams, and resilience training for high-stress situations should, to align with the reality of everyday healthcare settings, encompass the response of the entire team, not just individual team members. Moreover, to date, cited studies have focused only on stages 1 and 2 at the individual level. However, we should not rule out the possibility that the other stages may need to be activated at any time to address students' needs.

Recently, Krogh et al. [37] summarized the main expectations that students have for dealing with errors in clinical practice, including more knowledge about contributing factors, strategies to tackle them, attention to learning needs and wishes for the future healthcare system. They have identified as trigger of the second victim syndrome the severity of patient-injury and that the AE be completely unexpected.

## Implications for trainers & Health Policy

Collaboration among faculty, mentors, health disciplines students, and healthcare institutions is vital for promoting a learning culture that avoids blame, punishment, and shame and fear which will benefit the quality that patients received. This approach makes speak-up more straightforward, allowing continuous improvement in patient safety by installing a learning from errors culture. Ensuring safe practices requires close cooperation between the university and healthcare institutions [57]. Several practical implications of this study are summarized in Supplementary Table 4.

Psychological traumatizing events such as life-threating events, needle sticks, dramatic deaths, violent and threatening situations, torpid patient evolution, resuscitations, complaints, suicidal tendencies, and harm to patients are in the daily bases of healthcare workers. Errors occur all too frequently in the daily work of healthcare professionals. It is not just a matter of doctors or nurses, but it affects all healthcare workers. Ensuring their resilience in these situations should be a fundamental part of their training. This can be achieved through simulation exercises within the context of clinical practices, as it should be one of the key educational objectives. Specifically, clinical mistakes often have a strong emotional impact on professionals, and it seems that students (future professionals) are not receiving the necessary training to cope with the realities of clinical practice. Furthermore, during their training period, they may be affected by witnessing the consequences of AEs experienced by patients, which can significantly influence how professionals approach their work (e.g., defensive practices) [58] and their overall experience (e.g., detachment) [59]. There are proposals for toolkits that have proven to be useful [31, 60], and the data clearly indicate that educators should not delay further including educational content for their students to deal with errors and other highly stressful situations in healthcare practice [52]. Adapting measures within the academic environment and at healthcare facilities that host students in training programs is a task that we should no longer postpone.

## Future research directions

Individual differences in reactions to stress can modulate the future performance of current students and condition their resilience capacity [61]. This aspect should be studied in more detail alongside gender bias regarding mistakes made by man and woman [62]. The student perception of psychological safety to speak openly with their mentors [63], is also a crucial aspect in this training phase. Additionally, their conceptualization of human fallibility [63, 64] needs to be analyzed to identify the most appropriate educational contents.

Both witnessing errors with serious consequences and being involved in them can affect their subsequent professional development. Analyzing the impact of these incidents to prevent inappropriate defensive practices or dropouts requires greater attention. Future studies could link these experiences to attitudes towards incident reporting and open disclosure with patients.

## Limitations of the study

This review was limited to publications available in selected databases and might be subject to publication bias. The selection of studies could have been biased by the search strategy (controlled using a very broad strategy) or by the databases selected (controlled by choosing the four most relevant databases). Despite employing a comprehensive search strategy, relevant studies not indexed in the chosen databases may have been omitted. In the case of three articles, access to the full text was not available. There were no language limitations since there was no restructuring of the search. On the other hand, selection bias was controlled because the review was carried out by independent parties and with a third party for discrepancies. Regarding the results, the included studies exhibited considerable variability in design, interventions, and outcomes. This heterogeneity reflects the diverse educational settings and methodologies employed to address the second victim phenomenon but limits the generalizability of findings. In addition, most of the studies were conducted in high-income countries, which may not reflect the experiences or interventions applicable in low- and middle-income settings.

In conclusion, students also undergo the second victim experience, which may impact their future professional careers and personal lives. Interventions aimed at training healthcare discipline students to address the emotional experience of being involved in or witnessing mistakes causing harm to patients during their clerkships are currently scarce, scattered, and do not yield conclusive results on their effectiveness. Furthermore, most studies have focused on medical and nursing students, neglecting other healthcare disciplines such as pharmacy or psychology.

Despite some positive experiences, the provision of this type of training remains limited. There is a need for greater attention in the academic and clinical settings to identify students' needs and adequately prepare them for psychologically traumatizing events that occur frequently attending complex patients.

Efforts to support students in dealing with witnessing errors and highly stressful situations in clinical practice are essential to ensure their resilience and well-being of the future generation of healthcare professionals and ensure patient safety.

### Supplementary Information

**Supplementary Material 1.**

## Data Availability

The authors verify that the data supporting the conclusions of this study can be found in the article and its supplementary materials. However, data regarding the quality assessment process can be obtained from the corresponding author upon a reasonable request.
