# Supplementary material for: Unveiling the hidden struggle of healthcare students as second victims through a systematic review
Source: BMC Med Educ. 2024 Apr 8;24:378. doi: 10.1186/s12909-024-05336-y (PMC11000311; doi:10.1186/s12909-024-05336-y)
Supplement: Supplementary file 1 — Supplementary Material 1. [file 12909_2024_5336_MOESM1_ESM.docx]

Supplemental Material 1. Keywords and search strategy.

**Selection of Keywords following PICO method:**

- To define de study population:

Students

Trainees

Residents

Baccalaureate

Internship and Residency*

Students, Medical*

Second victims

Medical complications

Surgical complications

Errors

Mistakes

near-misses

clinical errors

medical errors

Patient safety incident (PSI)

- To define the intervention and comparator (if applicable):

Intervention

Support

Coping

support strategies

- To define the outcome:

Impact of adverse event

Symptoms

Adaptation

Psychological

Anxiety

Physicians / psychologists*

Patient Safety*

**Search strategy**

**Database:** PUBMED

August 5, 2023

| #1 | ((((((Student*[Title/Abstract]) OR (Trainee*[Title/Abstract])) OR (resident*[Title/Abstract])) OR (baccalaureate*[Title/Abstract])) OR (students, medical[MeSH Terms])) OR (internship and residency[MeSH Terms]) | [643,571](https://pubmed.ncbi.nlm.nih.gov/?term=%28%28%28%28%28%28Student%2A%5BTitle%2FAbstract%5D%29+OR+%28Trainee%2A%5BTitle%2FAbstract%5D%29%29+OR+%28resident%2A%5BTitle%2FAbstract%5D%29%29+OR+%28baccalaureate%2A%5BTitle%2FAbstract%5D%29%29+OR+%28students%2C+medical%5BMeSH+Terms%5D%29%29+OR+%28internship+and+residency%5BMeSH+Terms%5D%29&sort=) |
| --- | --- | --- |
| #2 | ((((("second victim"[Title/Abstract]) OR ("Patient safety incident*"[Title/Abstract])) OR ("medical error*"[Title/Abstract])) OR ("clinical error*"[Title/Abstract])) OR (medical mistake[MeSH Terms])) OR ("medical mistake"[Title/Abstract]) | [125,524](https://pubmed.ncbi.nlm.nih.gov/?term=%28%28%28%28%28%22second+victim%22%5BTitle%2FAbstract%5D%29+OR+%28%22Patient+safety+incident%2A%22%5BTitle%2FAbstract%5D%29%29+OR+%28%22medical+error%2A%22%5BTitle%2FAbstract%5D%29%29+OR+%28%22clinical+error%2A%22%5BTitle%2FAbstract%5D%29%29+OR+%28medical+mistake%5BMeSH+Terms%5D%29%29+OR+%28%22medical+mistake%22%5BTitle%2FAbstract%5D%29&sort=) |
| #3 | (((((("support"[Title/Abstract]) OR ("coping"[Title/Abstract])) OR ("impact of adverse event"[Title/Abstract])) OR (Physicians / psychology*[Title/Abstract])) OR (Psychological[Title/Abstract])) OR (anxiety[Title/Abstract])) OR (adaptation[Title/Abstract]) | [1,926,883](https://pubmed.ncbi.nlm.nih.gov/?term=%28%28%28%28%28%28%22support%22%5BTitle%2FAbstract%5D%29+OR+%28%22coping%22%5BTitle%2FAbstract%5D%29%29+OR+%28%22impact+of+adverse+event%22%5BTitle%2FAbstract%5D%29%29+OR+%28Physicians+%2F+psychology%2A%5BTitle%2FAbstract%5D%29%29+OR+%28Psychological%5BTitle%2FAbstract%5D%29%29+OR+%28anxiety%5BTitle%2FAbstract%5D%29%29+OR+%28adaptation%5BTitle%2FAbstract%5D%29&sort=) |
| #4 | **#1 AND #2 AND #3** | 663 |
| #5 | #4 NOT "fatigue"[Title/Abstract] | 641 |
| #6 | #5 NOT "burnout"[Title/Abstract] | 590 |
| #7 | #6 NOT "validation"[Title/Abstract] | 574 |
| #8 | #7 NOT (congress[Filter] OR consensusdevelopmentconference[Filter] OR consensusdevelopmentconferencenih[Filter] OR editorial[Filter] OR guideline[Filter] OR letter[Filter] OR meta-analysis[Filter] OR practiceguideline[Filter] OR review[Filter] OR systematicreview[Filter]) | 531 |

**Database:** EMBASE

August 5, 2023

| **#1** | 'student'/mj OR 'student*':ti,ab OR 'baccalaureate nursing student'/mj OR 'baccalaureate*':ti,ab OR 'trainee*':ti,ab OR 'resident'/mj OR 'resident*':ti,a | 1002026 |
| --- | --- | --- |
| **#2** | 'second victim'/exp OR 'second victim' OR 'second victim*':ab,ti OR 'error'/exp OR 'error' OR 'error*':ab,ti OR 'mistake*':ab,ti OR 'patient safety incident*':ab,ti OR 'near miss (health care)'/exp OR 'near miss (health care)' OR 'near-miss*':ab,ti OR 'medical complication*':ab,ti OR 'surgical complication*':ab,ti OR 'adeverse event*':ab,ti | 934319 |
| **#3** | 'intervention*':ab,ti OR 'support*':ab,ti OR 'coping*':ab,ti | 4123317 |
| **#4** | 'emotional*':ab,ti OR 'psychological*':ab,ti OR 'impact of adverse event':ab,ti OR 'anxiety':ab,ti | 899419 |
| **#5** | #1 AND #2 AND #3 AND #4 | 602 |
| **#6** | #1 AND #2 AND #3 AND #4 NOT ('fatigue':ab,ti OR 'burnout':ab,ti OR 'validation':ab,ti) | 489 |
| **#7** | #6 NOT ('case report'/de OR 'meta analysis'/de OR 'nonhuman'/de OR 'practice guideline'/de OR 'systematic review'/de OR 'validation process'/de OR 'validation study'/de) AND ('conference abstract'/it OR 'conference paper'/it OR 'conference review'/it OR 'note'/it OR 'review'/it OR 'tombstone'/it) | 465 |

**Database:** Psycinfo

August 5, 2023

| S1 | TI student* OR AB student* OR TI trainee* OR AB trainee* OR TI resident* OR AB resident* OR MA (internship and residency) OR TI baccalaureate* OR AB baccalaureate* | 665,154 |
| --- | --- | --- |
| S2 | TI second victim* OR AB second victim* OR TI medical error* OR AB medical error* OR TI clinical error* OR AB clinical error* OR TI medical mistake* OR AB medical mistake* OR TI Patient safety incident* OR AB Patient safety incident* | 2,729 |
| S4 | TI support* OR AB support* OR TI coping OR AB coping OR TI impact of adverse event OR AB impact of adverse event OR TI anxiety OR AB anxiety OR TI adaptation OR AB adaptation OR TI psycholog* OR AB psycholog* |  |
| S5 | S1 AND S2 AND S4 | 165 |
| S6 | S5 NOT TI fatigue NOT AB fatigue NOT TI burnout NOT AB burnout NOT TI validation NOT AB validation | 146 |

**Database:** Scopus

August 5, 2023

( ( TITLE-ABS-KEY ( student* ) OR TITLE-ABS-KEY ( trainee* ) OR TITLE-ABS-KEY ( resident* ) OR TITLE-ABS-KEY ( baccalaureate* ) OR TITLE-ABS-KEY ( "internship and residency" ) ) AND ( TITLE-ABS-KEY ( "second victim*" ) OR TITLE-ABS-KEY ( "Patient safety incident*" ) OR TITLE-ABS-KEY ( "medical error*" ) OR TITLE-ABS-KEY ( "clinical error*" ) OR TITLE-ABS-KEY ( "medical mistake*" ) ) AND ( TITLE-ABS-KEY ( "support" ) OR TITLE-ABS-KEY ( "coping" ) OR TITLE-ABS-KEY ( "impact of adverse event" ) OR TITLE-ABS-KEY ( "anxiety" ) OR TITLE-ABS-KEY ( "adaptation" ) OR TITLE-ABS-KEY ( psycholog* ) ) ) AND NOT ( TITLE-ABS-KEY ( fatigue ) OR TITLE-ABS-KEY ( burnout ) OR TITLE-ABS-KEY ( validation ) ) AND ( EXCLUDE ( DOCTYPE , "no" ) OR EXCLUDE ( DOCTYPE , "ed" ) OR EXCLUDE ( DOCTYPE , "le" ) OR EXCLUDE ( DOCTYPE , "sh" ) OR EXCLUDE ( DOCTYPE , "ch" ) OR EXCLUDE ( DOCTYPE , "bk" ) OR EXCLUDE ( DOCTYPE , "cp" ) ) AND ( EXCLUDE ( SRCTYPE , "k" ) )

N= 480

**Total: 1622**

Supplementary Table 1. Quality evaluation of selected quasi-experimental studies (n=8).

|  | Cause and effect variables | Participants from the compared groups | Other exposures/treatments occurring in the same time | Control group | Multiple measurements of the outcomes | Complete follow-up | Outcomes measured in the same way | Outcomes measured in a reliable way | Statistical analysis |
| --- | --- | --- | --- | --- | --- | --- | --- | --- | --- |
| Breslin 2019^3^ | √ | √ | √ | x | x | ? | √ | √ | √ |
| Davis 2020^9^ | √ | √ | √ | x | x | √ | √ | √ | √ |
| Gillies 2011^12^ | √ | √ | √ | x | x | √ | √ | ? | x |
| Kim 2017^21^ | √ | √ | √ | x | x | √ | √ | ? | √ |
| Mohsin 2019^33^ | √ | √ | √ | x | x | √ | √ | ? | x |
| Musunur^35^ | √ | √ | √ | x | x | √ | √ | ? | √ |
| Roh 2014^46^ | √ | √ | √ | x | x | √ | √ | ? | √ |
| Ryder^47^ | √ | x | √ | √ | x | ? | √ | ? | √ |

√: Yes; x: No󠆩; 󠆩 󠆩?: Unclear; NA: Not applicable

Supplementary Table 2. Quality evaluation of selected Cross-sectional studies (n=2)

|  | Clear inclusion criteria | Detailed description of subjects and setting | Exposure measured in a valid and reliable way | Standard criteria used for measurement of the condition | Confounding factors identified | Strategies to deal with confounding factors | Outcomes measured in a valid and reliable way | Appropriate statistical analysis |
| --- | --- | --- | --- | --- | --- | --- | --- | --- |
| Le 2020^25^ | ? | ? | ? | ? | ? | ? | ? | ? |
| Rinaldi 2022^45^ | √ | √ | √ | √ | ? | ? | √ | √ |

√: Yes; x: No󠆩; 󠆩 󠆩?: Unclear; NA: Not applicable

Supplementary Table 3. Quality evaluation of selected Qualitative studies (n=8)

|  | Congruity between philosophical perspective and methodology | Congruity between methodology and the objectives | Congruity between methodology and methods used to collect data | Congruity between methodology and representation and analysis of data | Congruity between methodology and the interpretation | Statement locating the researcher culturally or theoretically | Influence of the researcher on the research | Participants adequately represented | Ethical research | Conclusions report flow from the data analysis |
| --- | --- | --- | --- | --- | --- | --- | --- | --- | --- | --- |
| Browall Krogh 2023^22^ | √ | √ | √ | √ | √ | ? | √ | x | √ | √ |
| Hanson 2019^15^ | √ | √ | √ | √ | √ | ? | √ | x | √ | √ |
| Huang 2020^16^ | √ | √ | √ | √ | √ | x | √ | x | √ | √ |
| Lane 2021^24^ | √ | √ | √ | √ | √ | x | ? | x | √ | √ |
| Moushino Tavares 2022^34^ | √ | √ | √ | √ | √ | x | x | ? | √ | √ |
| Noland 2015^36^ | √ | √ | √ | √ | √ | x | x | √ | √ | √ |
| Thomas 2015^56^ | √ | √ | √ | √ | √ | x | x | ? | √ | √ |
| Zieber 2015^61^ | √ | √ | √ | √ | √ | x | √ | x | √ | √ |

√: Yes; x: No󠆩; 󠆩 󠆩?: Unclear; NA: Not applicable

Supplementary Table 4. Actions that could be implemented in academic and healthcare settings.

| **Stakeholders** | **Actions** |
| --- | --- |
| Deans and directors of faculties and schools, heads of vice-rectorates responsible for student affairs, and executives of healthcare institutions | Protecting the mental wellbeing of healthcare students is imperative, considering their future contribution to delivery of a safe healthcare system.  Developing protocols to guide students on actions following an adverse event, addressing patient needs and rights, as well as students' emotional state. |
| Academic and practical mentors | Understanding and addressing the emotional and psychological implications of adverse events on students.  Implementing methods and interventions to support students in reporting errors. |
| Curriculum activity planners | Since healthcare students can become second victims, it is essential to prepare them to cope with this phenomenon. Incorporate curriculum content that addresses how to cope with highly stressful situations commonly encountered in professional practice. |
| Technical staff involved in designing accreditation and quality programs for university education | Incorporate standards regarding the response educational institutions provide when an adverse event occurs due to a mistake by a student during their practical training. |
| Insurance companies covering risk and personal liability for actions during the course of university education and those managing professional liability insurance policies | The insurance policy coverage could encompass compensation and patient care needs, along with emotional support for affected students.  Facilitating platforms where insurers can share experiences and best practices with academic institutions to enhance responses for students when they become second victims. |
